# Association of hepatitis status with surgical outcomes in patients with dual hepatitis B and C related hepatocellular carcinoma

**DOI:** 10.1186/s13027-017-0137-6

**Published:** 2017-05-25

**Authors:** Xiu-Tao Fu, Ying-Hong Shi, Jian Zhou, Yuan-Fei Peng, Wei-Ren Liu, Guo-Ming Shi, Qiang Gao, Xiao-Ying Wang, Kang Song, Jia Fan, Zhen-Bin Ding

**Affiliations:** 1 Liver Cancer Institute, Zhongshan Hospital, and Key Laboratory of Carcinogenesis and Cancer Invasion (Ministry of Education), Fudan University, Shanghai, 200032 China; 2 Institute of Biomedical Sciences, Fudan University, Shanghai, 200032 China; 3 Department of Liver Surgery, Zhongshan Hospital, Fudan University, 1609 Xietu Road, Shanghai, 200032 China

**Keywords:** Hepatocellular carcinoma, Hepatitis B, Hepatitis C, Surgical outcomes

## Abstract

**Background:**

The conception that serological hepatitis markers determined surgical prognosis of hepatocellular carcinoma (HCC) associated with hepatitis B (HBV) or hepatitis C (HCV) has been well defined. However, little is known about the relationship between surgical outcomes and serological hepatitis markers in patients with dual HBV and HCV related HCC.

**Methods:**

A retrospective analysis of the clinical data of 39 HCC patients with HBV-HCV coinfection who underwent curative hepatectomy between 2001 and 2011 was performed. HBV DNA quantification, expression of HBV antigens, anti-HCV signal-to-cutoff ratio (S/CO) and some clinicopathological characteristics were investigated to show the potential relationship among them and the surgical prognosis.

**Results:**

The Cox proportional hazards model identified that HBV DNA quantification of 1,000 IU/mL or higher, HBeAg seropositivity, tumor size of greater than 5 cm, multiple tumors, and vascular invasion were risk factors for HCC prognosis. Thus, HBV DNA quantification, HBsAg level, HBeAg status and HCV-Ab level which may reveal the hepatitis status were further analyzed. The overall survival time in the group with high (≥1,000 IU/mL) HBV DNA quantification was significantly lower than the group with low (<1,000 IU/mL) HBV DNA quantification. Similarly, the high HBsAg level (≥1,000 IU/mL) was associated with poor survival compared with the low HBsAg level. Moreover, HBeAg seropositivity determined a higher cumulative risk for death. However, no significant difference was observed in overall survival time between the groups with low (<10.9 S/CO) and high (≥10.9 S/CO) HCV-Ab level. Compared to HCV-Ab high-level group, the serological HBsAg level was observed significantly higher in HCV-Ab low-level group. Furthermore, the data we analyzed showed these 4 serological hepatitis markers were not correlated with cumulative recurrence rate. On multivariate analysis, none of serological hepatitis markers was an independent prognostic factor for HCC patients with dual hepatitis B and C.

**Conclusion:**

Among HCC patients with HBV-HCV coinfection, those who with preoperatively high HBV DNA quantification or HBeAg seropositivity had a short survival time and served as poor survival indicators. Serological expression of HBV status rather than HCV status might potentially dominate the surgical outcomes of the Chinese HCC patients with HBV-HCV coinfection.

**Electronic supplementary material:**

The online version of this article (doi:10.1186/s13027-017-0137-6) contains supplementary material, which is available to authorized users.

## Background

Hepatocellular carcinoma (HCC) accounts for the second place of the cancer related death [1] and 70–85% of the total liver cancer burden [2] in the world. Curative hepatic resection predominates in the treatment for HCC although several novel treatment options have been applied in the clinical practice in the past few decades [3, 4]. With the recent advances in medicine and technique, overall post-hepatectomy survival rate of HCC patients increased in recent years [5]. Thus, it is extremely important to discover the risk factors for HCC surgical outcomes.

The major risk factors of HCC include alcoholism, cirrhosis, viral hepatitis and fatty liver diseases [2, 6–8]. Hepatitis B virus (HBV) and hepatitis C virus (HCV) are considered to be the main etiological factors for HCC. These two infectious agents are estimated to be responsible for 78% of the HCC cases in the world [9]. HBV-HCV coinfection is thought to be frequent in occurrence, especially in endemic areas, since the virus shares modes of transmission [10].

A retrospective case–control study showed that, compared with non-HBV and non-HCV HCC patients, HBV-HCC patients had significantly worse pre- and postoperative liver function and significantly worse overall survival (OS) and recurrence-free survival (RFS) rates after hepatectomy [11]. Compared with HBV-HCC, HCV-HCC tends to be less differentiated, and to have a higher incidence of vascular invasion and synchronous multicentric recurrence than other HCC types. In addition, HCV-positive livers are more likely to be cirrhotic, have worse liver function, and to be classified as Child B or C which may impact the prognosis of the patients [12, 13].

However, less is known about the clinical characteristics and outcomes of HCC patients with HBV-HCV coinfection. In this retrospective study, we would like to present a detailed clinical data analysis along with their clinic-pathological features. The primary aim of our report was to explore the association between hepatitis status and HCC surgical outcomes in patients with HBV-HCV dual infection which may help to improving the postoperative prognosis.

## Methods

### Study Patients

From 2001 to 2011, a total of 39 patients with chronic HBV and HCV dual infection who underwent curative partial hepatectomy at Liver Cancer Institute, Zhongshan Hospital, Fudan University, China and postoperative pathologically diagnosed as hepatocellular carcinoma were collected in our study. Ethical approval was obtained from the Zhongshan Hospital Research Ethics Committee, and written informed consent was obtained from each patient.

### Data Collection

All patients underwent serological testing 1 week before surgery to determine the hepatitis B surface antigen (HbsAg), hepatitis B e antigen (HbeAg), the α-fetoprotein (AFP) level and liver biochemical tests. Due to technical limitations, 20 patients did not receive quantitative determination of serum HBV-DNA load, and 19 patients only received qualitative detection of HCV-Ab. From 2007, the quantitative determination of HBV-DNA load and HCV-Ab were adopted.

### Clinic-pathological Charicteristics

Clinicopathological characteristics in this study were selected for their potential relation to the prognosis on the basis of the previous studies, including age (≤52 vs >52 years), gender (male vs female), serum AFP concentration (≤20 vs >20 ng/mL), HBsAg level (<1000 vs ≥1000 IU/mL), HBeAg status (positive vs negative), HBV-DNA level (<1000 vs ≥1000 IU/mL), HCV-Ab level (<10.9 S/CO vs ≥10.9 S/CO), severity of cirrhosis (yes vs no), tumor size (≤5 vs >5 cm), number of tumor nodules (single vs multiple), tumor capsule (yes vs no), vascular invasion (yes vs no), differentiation of tumor cells (Edmondson’s Classification I/II vs III/IV [14]). The clinicopathological characteristics of the patients were summarized in Table 1.

**Table 1:** Clinic-pathological characteristics and univariate analysis of factors associated with OS and RFS

		OS			RFS		
Variable	*n*	HR	95% CI	*P* value	HR	95% CI	*P* value
Sex (female vs male)	5 vs 34	1.179	0.345–4.031	0.792	1.016	0.229–4.509	0.984
Age years (≤52 vs >52)	21 vs 18	0.754	0.312–1.824	0.531	1.680	0.596–4.732	0.326
AFP (ng/ml; ≤20 vs >20)	13 vs 25	1.734	0.629–4.778	0.287	1.214	0.414–3.555	0.724
HBsAg (IU/mL; <1000 vs ≥1,000)	6 vs 28	5.915	0.784–44.637	0.085	1.561	0.340–7.179	0.567
HBeAg (negative vs positive)	33 vs 6	8.931	2.661–29.982	0.000	3.361	0.848–13.321	0.085
HBV-DNA (IU/mL; <1000 vs ≥1,000)	10 vs 9	7.798	1.580–38.496	0.012	2.808	0.647–12.186	0.168
HCV-Ab (S/CO; <10.9 vs ≥10.9)	8 vs 12	0.579	0.152–2.207	0.424	0.808	0.189–3.455	0.774
Liver cirrhosis (no vs yes)	13 vs 26	1.245	0.478–3.243	0.654	2.118	0.596–7.519	0.246
Tumor size (cm; ≤5 vs >5)	25 vs 14	4.336	1.753–10.722	0.001	2.302	0.793–6.686	0.125
Tumor number (single vs multiple)	31 vs 8	3.155	1.145–8.695	0.026	1.679	0.455–6.190	0.437
Vascular invasion (no vs yes)	26 vs 13	3.352	1.358–8.272	0.009	3.806	13.60–10.646	0.011
Capsule (no vs yes)	12 vs 26	1.319	0.474–3.670	0.596	0.775	0.259–2.317	0.648
Tumor differentiation (I-II vs III-IV)	21 vs 16	0.798	0.308–2.068	0.642	0.664	0.221–1.992	0.465

Note: Univariate analysis, Cox proportional hazards regression model

Abbreviations: *HR*, Hazard ratio; 95% CI, 95% confidence interval; *AFP*, alpha-fetoprotein; *HCV-Ab*, HCV antibody

### Follow-Up

Follow-up was completed in June 15, 2016. Data were obtained at last follow-up for patients without relapse or death. As described in our previous study [15], all patients were monitored prospectively by serum AFP, abdomen ultrasonography, and chest x-ray every 1 to 6 months according to the postoperative time. For patients with test results suggestive of recurrence, computed tomography and/or magnetic resonance imaging were used to verify whether recurrence had occurred. A diagnosis of recurrence was based on typical imaging appearance in computed tomography and/or magnetic resonance imaging scan and an elevated AFP level. OS time was defined as the time period from the date of surgery to the confirmed death date for dead patients or from the date of surgery to the date of last follow-up for surviving patients. RFS was defined as the time period from the date of surgery to confirmed tumor relapse date for relapsed patients or from the date of surgery to the date of last follow-up for nonrecurrent patients.

### Statistical Analyses

Patient OS and RFS rates after surgical resection were calculated using the Kaplan–Meier method. A Chi-square test or Fisher’s exact test was performed to compare qualitative variables. The risk factors of OS and RFS after surgery were evaluated by the univariate and the multivariate Cox proportional hazards models. The variables of the multivariate analysis were determined if their *P* values were less than 0.05 during the univariate analysis. The forward LR method was adopted during the multivariate analysis to avoid the multicollinearity. The *P* value for a two-tailed test of less than 0.05 was considered statistically significant. All statistical analyses were performed using SPSS 22.0 for Windows (IBM, Chicago, IL).

## Results

### Overall survival and Recurrence-free survival

From 2001 to 2011, a total of 39 patients with chronic HBV and HCV dual infection who underwent curative hepatectomy at our institute were included in this study. Their postoperative pathological diagnosis was confirmed to be hepatocellular carcinoma. The median overall survival time was 50.1 months and the postoperative 1-, 3-, and 5-year overall survival rates of these patients was 89.6%, 73.3%, and 55.9%, respectively. Afterwards, the median recurrence-free survival time was 45.0 months and the postoperative 1-, 3-, and 5-year recurrence-free survival rates of them was 86.8%, 69.1%, and 53.2%, respectively.

### HBV infection status and patient survival

**Fig. 1:**
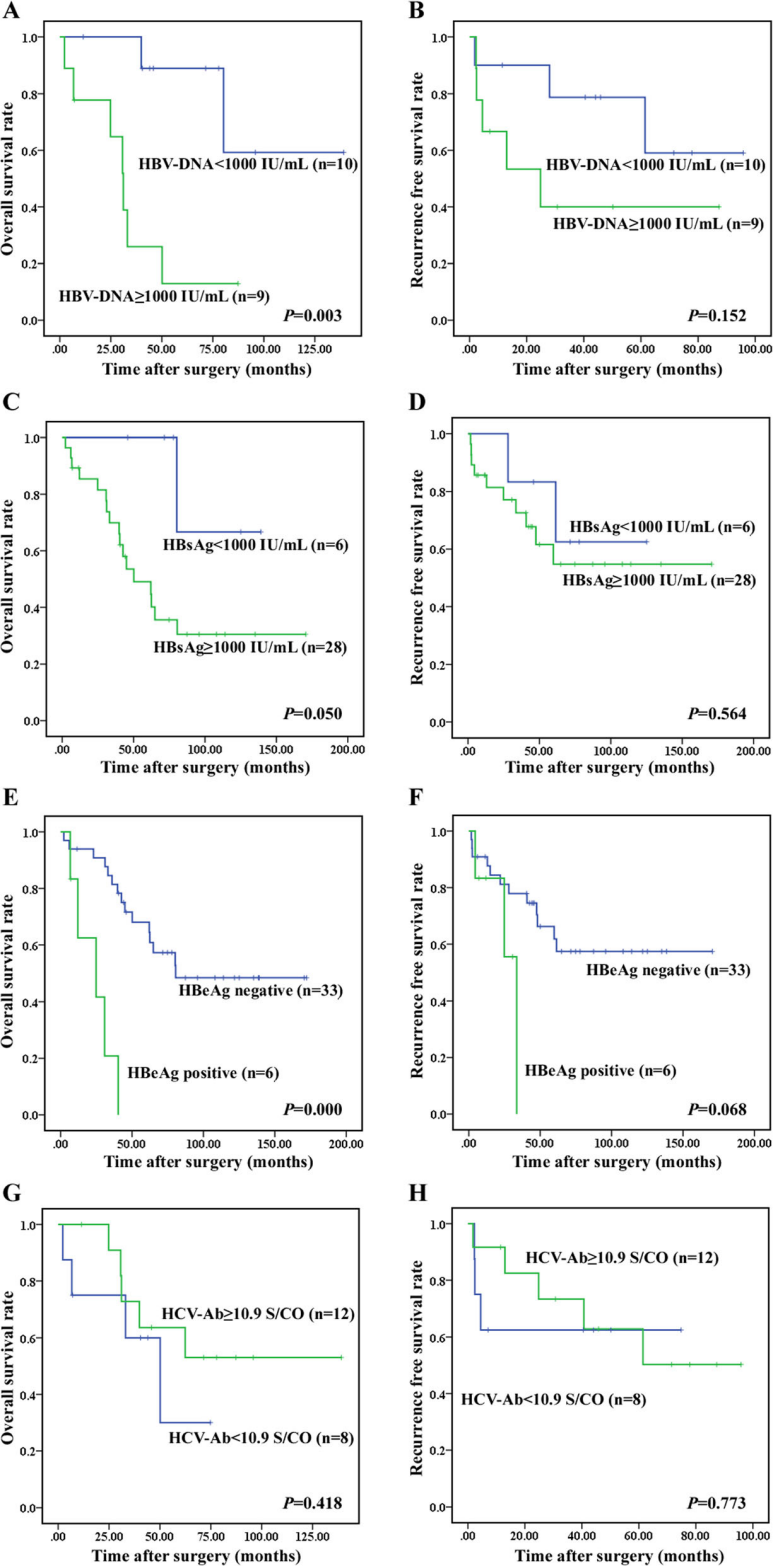
Kaplan-Meier survival analysis of hepatitis markers and HCC patients with dual hepatitis B and C. **a**, OS rates between high HBV-DNA level (≥1000 IU/mL, *n* = 9) group and low HBV-DNA level (<1000 IU/mL, *n* = 10); *P* = 0.003; **b**, RFS rates between high HBV-DNA level (≥1000 IU/mL, *n* = 9) group and low HBV-DNA level (<1000 IU/mL, *n* = 10); *P* = 0.152; **c**, OS rates between high HBsAg level (≥1000 IU/mL, *n* = 28) group and low HBsAg level (<1000 IU/mL, *n* = 6); *P* = 0.050; **d**, RFS rates between high HBsAg level (≥1000 IU/mL, *n* = 28) group and low HBsAg level (<1000 IU/mL, *n* = 6); *P* = 0.564; **e**, OS rates between HBeAg positive group (*n* = 6) and HBeAg negative group (*n* = 33); *P* = 0.000; **f**, RFS rates between HBeAg positive group (*n* = 6) and HBeAg negative group (*n* = 33); *P* = 0.068; **g**, OS rates between high HCV-Ab level (≥10.9 S/CO, *n* = 12) group and low HCV-Ab level (<10.9 S/CO, *n* = 8); *P* = 0.418; **h**, RFS rates between high HCV-Ab level (≥10.9 S/CO, *n* = 12) group and low HCV-Ab level (<10.9 S/CO, *n* = 8); *P* = 0.773

Kaplan-Meier survival estimates and the log-rank test were used to calculate the factors associated with the OS and RFS for all the patients. Interestingly, OS but not the RFS, was significantly associated with HBV DNA load, HBsAg level and HBeAg status. The overall survival time in the group with high (≥1000 IU/mL) HBV DNA quantification was significantly lower than the group with low (<1000 IU/mL) HBV DNA quantification (34.33 ± 8.63 vs 110.65 ± 16.50 months; *P* = 0.003, Fig. 1a). Similarly, the high HBsAg level (≥1000 IU/mL) was associated with poor survival compared with the low HBsAg level (79.45 ± 12.88 vs 119.49 ± 16.01 months; *P* = 0.050, Fig. 1c). Moreover, HBeAg seropositivity determined a higher cumulative risk for death (23.59 ± 5.89 vs 107.40 ± 12.07 months; *P* = 0.000, Fig. 1e). Therefore, HBV-DNA, HBsAg and HBeAg which represent the preoperational HBV status impacts OS after curative hepatic resection in these patients.

### HCV infection status and patient survival

HCV-Ab S/CO ratio was found to be highly accurate at predicting HCV viremia. And at an anti-HCV S/CO ratio cutoff value of 10.9, sensitivity and specificity were high [16]. As a result, we selected 10.9 S/CO as the cutoff level for HCV-Ab and categorized these patients into two groups. However, no significant difference was observed in OS and RFS between the groups with low (<10.9 S/CO) and high (≥10.9 S/CO) HCV-Ab level (OS: 43.56 ± 10.32 vs 91.89 ± 15.64 months, *P* = 0.418; RFS: 47.88 ± 12.28 vs 63.797 ± 10.96 months, *P* = 0.773, Fig. 1g, h).

### HCV-Ab level and HBsAg level

Previous cross-sectional and in vitro studies have suggested that HCV coinfection has an inhibitory effect on HBV replication [17, 18], but the in vivo data do not support it [19, 20]. In this study, quantitative analysis indicated that the level of HBsAg was significantly higher in group with low HCV-Ab (<10.9 S/CO) level than in group with high (≥10.9 S/CO) HCV-Ab level (6696.75 ± 1521.16 vs 3221.99 ± 3104.90; *P* = 0.004).

### Hepatitis status and tumor features

A comparison of hepatitis status (HBsAg, HBeAg, HBV-DNA, and HCV-Ab) between tumor features (tumor size, vascular invasion and TNM stage) revealed that HBeAg-positive patients were more likely to have a larger tumor size (Chi-Square value = 4.712, *P* = 0.030). There was no significant difference between the other groups (Additional file 1: Table S1).

### Other clinicopathological characteristics and patient survival

**Fig. 2:**
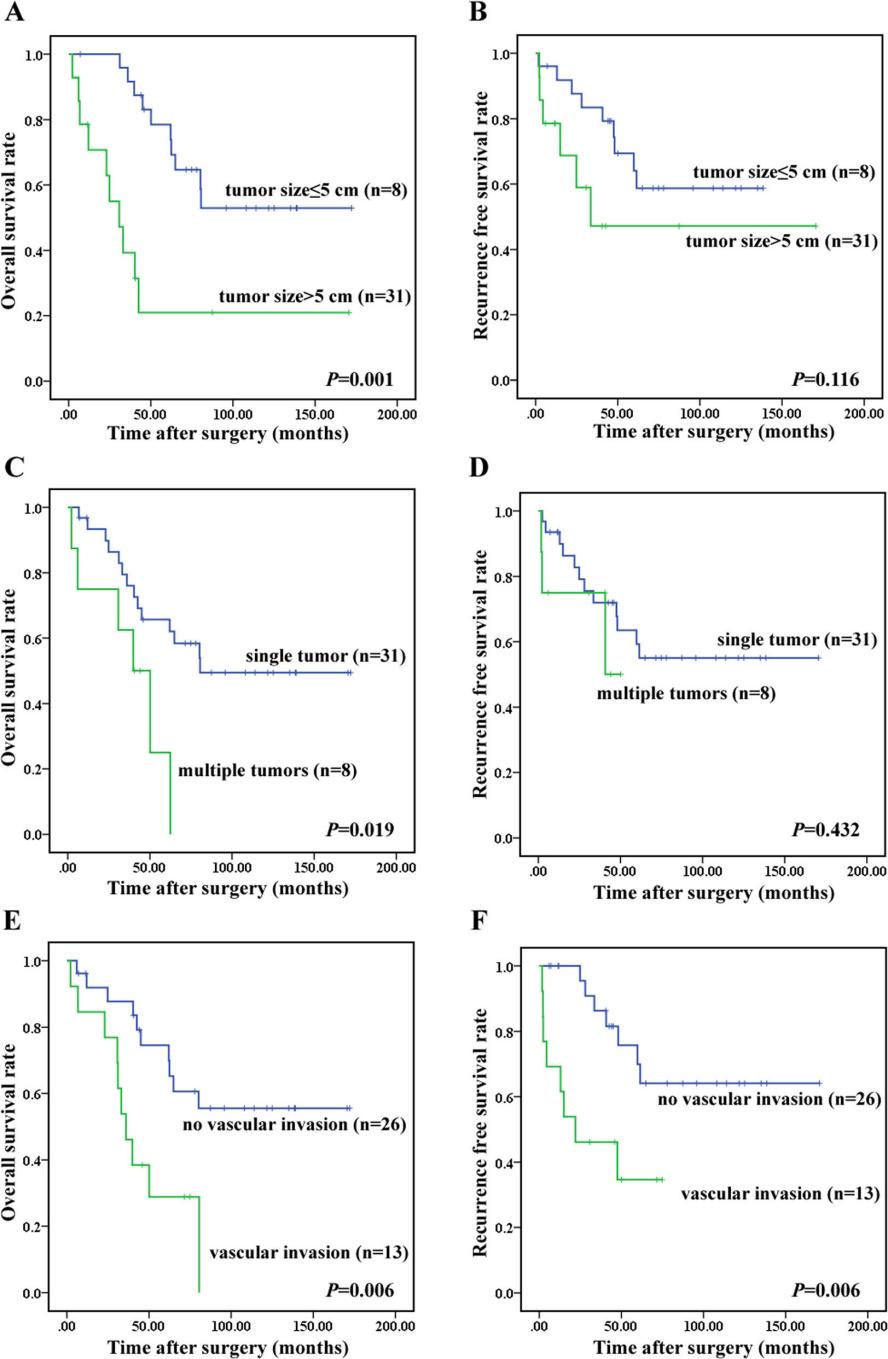
Kaplan-Meier survival analysis of clinic-pathological characteristics and HCC patients with dual hepatitis B and C. **a**, OS rates between large tumor (>5 cm, n = 8) group and small tumor (≤5 cm, n = 31) group; *P* = 0.001; **b**, RFS rates between large tumor (>5 cm, *n* = 8) group and small tumor (≤5 cm, *n* = 31) group; *P* = 0.116; **c**, OS rates between single tumor (*n* = 31) group and multiple tumors (*n* = 8) group; *P* = 0.019; **d**, RFS rates between single tumor (*n* = 31) group and multiple tumors (*n* = 8) group; *P* = 0.432; **e**, OS rates between no vascular invasion group (*n* = 26) and vascular invasion group (*n* = 13); *P* = 0.006; **f**, RFS rates between no vascular invasion group (*n* = 26) and vascular invasion group (n = 13); *P* = 0.006

Worse overall survival was found in association with tumor size of greater than 5 cm (54.13 ± 17.88 vs 118.01 ± 12.79 months; *P* = 0.001, Fig. 2a), multiple tumors (38.00 ± 8.65 vs 107.05 ± 12.62 months; *P* = 0.019, Fig. 2c) and vascular invasion (43.65 ± 7.86 vs 115.89 ± 13.61 months; *P* = 0.006, Fig. 2e). Not tumor size and number, but vascular invasion was significantly correlated with RFS (36.06 ± 8.76 vs 125.01 ± 13.97 months; *P* = 0.006, Fig. 2b, d, f).

### Multivariate logistic regression analysis

All variables with *P* < 0.05 in the univariate analysis were placed into the multivariate Cox regression model. As shown in Table 2, none of serological hepatitis markers was an independent prognostic factor for HCC patients with dual hepatitis B and C.

**Table 2:** Multivariate analysis of factors associated with OS

	OS		
Variable	HR	95% CI	*P* value
Tumor size (cm; ≤5 vs >5)	1.460	0.159–13.418	0.738
Tumor number (single vs multiple)	0.882	0.154–5.067	0.888
Vascular invasion (no vs yes)	7.612	0.771–75.141	0.082
HBeAg (negative vs positive)	7.503	0.494–113.848	0.146
HBV-DNA (IU/mL; <1000 vs ≥1,000)	3.235	0.378–27.714	0.284

## Discussion

Hepatocellular carcinoma is one of the most common cancers in China, with a relatively high mortality [6], and curative hepatic resection remains the common treatment in HCC patients. From a global perspective, viral hepatitis is the leading cause for HCC. It is critical to identify risk factors for the outcomes of HCC patients with viral hepatitis. In the present study, we investigated the association between hepatitis status and surgical outcomes in HCC patients with dual HBV-HCV infection.

HCC pathogenesis in HBV monoinfected patients has been studied extensively, and several important viral risk factors which indicate the HBV status have been identified, such as HBsAg level, seropositivity of HBeAg, high viral load. In our previous study [21], high HBsAg level (≥1000 IU/mL) is correlated with more aggressive tumor behavior and serves as a poor survival indicator in patients with surgically resected HBV-related HCC with low HBV load. Here, we continued to use the same standard in order to avoid the influence of HBV-DNA load. Our results demonstrated that the HBsAg level might be a potential risk factor for HCC in patients with dual HBV-HCV infection. HBV-DNA quantification is known to be significantly associated with decreased survival rate in HBV alone infected HCC patients [22]. In present study, similarly, the results demonstrated that high HBV load (≥1000 IU/mL) was correlated with poor surgical outcome of HCC patients with HBV-HCV coinfected. The presence of HBeAg was often used as a criterion for treatment before the introduction of HBV DNA examination [23]. It was reported at our institution that HBeAg seropositivity was an independent factor for overall survival in hepatitis B-related HCC patients after curative resection [24]. The similar conclusion was also identified in this study. Moreover, recent study revealed that HBeAg and its precursors promoted the progress of HCC by interacting with NUMB and decreasing p53 activity [25]. Therefore, in this study, HBeAg positive HCC patients usually had larger tumor compared to HBeAg negative patients. This finding provided some evidence for the association between HBV status and prognosis in HBV-HCV related HCC.

It was reported that the risk of developing HCC in patients with high anti-HCV Ab level is significantly higher than the risk in patients with low level [26]. In HCC patients with HCV monoinfection, recent study strongly suggests that the HCV-Ab level is a predictive factor for HCC recurrence, especially for late recurrence due to presumed multicentric carcinogenesis [27, 28]. And low HCV viral load predicted better long-term surgical outcomes in patients with HCC regardless of the serologic eradication of HCV [29]. Unfortunately, we found the HCV-Ab level was not associated with tumor recurrence or overall survival in HBV-HCV coinfected HCC patients. However, a relation between HBsAg level and anti-HCV Ab level was discovered in these patients. Due to the interaction between HCV and HBV infection, we tentatively put forward that HBV status may influence the replication of HCV and play a vital role in coinfected patients, especially in Chinese population. Consequently, further studies are needed to clarify the interaction between the two hepatitis viruses in vivo.

It is well known that specific tumor characteristics were also significantly associated with prognosis of HCC patients [30–33]. Similar to previous studies [34, 35], our research validated that large tumor size, multiple tumors and vascular invasion were significantly associated with poorer prognosis. However, as we can see, the selected serological hepatitis markers including HBsAg, HBeAg and HBV-DNA only correlate with OS, but not RFS. A potential explanation for this discrepancy may be attributed to the high percentage of cirrhosis in dual HBV-HCV infected HCC patients; a number of patients died of impaired liver function or cirrhosis during follow up, which did not allow them to develop multicentric tumor recurrence. However, liver stiffness measurement using elastography-based techniques and indocyanine green kinetics were not applied to quantitatively assess the severity of hepatic cirrhosis and function in our department until 2012, and further study was needed to interpret it.

There are some limitations to our study. First, this retrospective study only enrolled 39 coinfected patients from 2001 to 2011. And the quantitative determination of HBV-DNA and HCV-Ab was adopted to replace previous qualitative detection from 2006. Therefore, we only have HBV-DNA data of 19 cases and HCV-Ab level of 20 cases, which might affect the long-term clinical prognosis analysis. Second, two serological HBV markers (HBeAg and HBV-DNA) that have potential interaction were added into the multivariate analysis. As a result, when multivariate Cox regression model was performed, none of serological hepatitis markers was an independent prognostic factor in these patients. Finally, determination of serum HCV-RNA load which accurately evaluates the HCV load was not a routine procedure for admitted patient in our department before 2011.

In summary, serological expression of HBV status including HBsAg, HBeAg and HBV-DNA plays a predominant role in prediction of surgical survival in dual HBV and HCV related HCC patients. Our findings suggest that anti-HBV therapy could be a valid strategy for prolonged survival in coinfected HCC patients, especially in Chinese population.

## Conclusions

Our study shows that serological expression of HBV status rather than HCV status might potentially dominate surgical outcomes of the Chinese HCC patients with HBV-HCV coinfection. Anti-HBV therapy could be a valid strategy for prolonged survival in HBV-HCV coinfected HCC patients.

## Additional file

Additional file 1: Table S1. Comparison between hepatitis status and tumor features in HBV-HCV coinfection HCC patients. (DOCX 17kb)

## Abbreviations

HBeAg: Hepatitis B e Antigen
HBsAg: Hepatitis B Surface Antigen
HBV: Hepatitis B Virus
HCC: Hepatocellular Carcinoma
HCV: Hepatitis C Virus
HCV-Ab: HCV-Antibody
OS: Overall Survival
RFS: Recurrence-Free Survival
