# Supplementary material for: Association of hepatitis status with surgical outcomes in patients with dual hepatitis B and C related hepatocellular carcinoma
Source: Infect Agent Cancer. 2017 May 25;12:28. doi: 10.1186/s13027-017-0137-6 (PMC5445430; doi:10.1186/s13027-017-0137-6)
Supplement: Additional file 1: Table S1. — Comparison between hepatitis status and tumor features in HBV-HCV coinfection HCC patients. (DOCX 17kb) [file 13027_2017_137_MOESM1_ESM.docx]

**Table S1.** Comparison between hepatitis status and tumor features in HBV-HCV coinfection HCC patients

|  | Tumor size (cm; ≤5 vs >5) | | Vascular invasion (no vs yes) | | TNM (Stage I vs II/III) | |
| --- | --- | --- | --- | --- | --- | --- |
| Hepatitis status | Chi-Square Value | P Value | Chi-Square Value | P Value | Chi-Square Value | P Value |
| HBsAg (IU/mL; <1000 vs ≥1,000) | 2.758 | 0.097 | 0.000 | 1.000 | 0.885 | 0.347 |
| HBeAg (negative vs positive) | 4.712 | **0.030** | 0.000 | 1.000 | 0.997 | 0.318 |
| HBV-DNA (IU/mL; <1000 vs ≥1,000) | 2.534 | 0.111 | 1.295 | 0.255 | 1.760 | 0.185 |
| HCV-Ab (S/CO; <10.9 vs ≥10.9) | 0.078 | 0.780 | 0.208 | 0.648 | 0.000 | 1.000 |

**Note:** TNM staging was according to 7th edition of AJCC/UICC TNM Classification.
